# Recent advances in understanding how rod-like bacteria stably maintain their cell shapes

**DOI:** 10.12688/f1000research.12663.1

**Published:** 2018-02-28

**Authors:** Sven van Teeffelen, Lars D. Renner

**Affiliations:** 1 Department of Microbiology, Institut Pasteur, 75724 Paris Cedex 15, France; 2 Leibniz Institute of Polymer Research and the Max Bergmann Center of Biomaterials, 01069 Dresden, Germany

**Keywords:** bacterial functions, cell shape, cell size, bacterial metabolism, rod-like, E.coli, B.subtilis, bacterial physiology

## Abstract

Cell shape and cell volume are important for many bacterial functions. In recent years, we have seen a range of experimental and theoretical work that led to a better understanding of the determinants of cell shape and size. The roles of different molecular machineries for cell-wall expansion have been detailed and partially redefined, mechanical forces have been shown to influence cell shape, and new connections between metabolism and cell shape have been proposed. Yet the fundamental determinants of the different cellular dimensions remain to be identified. Here, we highlight some of the recent developments and focus on the determinants of rod-like cell shape and size in the well-studied model organisms *Escherichia coli* and *Bacillus subtilis*.

## Introduction

Shape and size of bacteria are important for many aspects of bacterial physiology, such as proper growth, motility, multi-cellularity, and host invasion ^1^. The wide range of diverse cell shapes and sizes in bacteria—from spherical to rod to even more bizarre star shapes covering 4 orders of magnitude in size—is astounding ^1, 2^. What is even more impressive is the precision of how bacterial cells control and maintain their cell shapes. Accordingly, cells have evolved the capacity to tightly regulate their own morphologies. Physically, cell shape is determined by the geometry of the peptidoglycan (PG) cell wall. The PG cell wall is a covalently bound meshwork of glycan strands that are interconnected by peptide bonds ^3, 4^. The glycan strands are likely oriented around the cell circumference in both Gram-negative and Gram-positive bacteria, which is supported by both cryo-electron tomography imaging ^5, 6^ and live-cell imaging of circumferential cell-wall insertion ^7– 9^. The PG meshwork mechanically resists the high turgor pressure and gives the cell its specific cell shape. As a consequence, without cell wall, bacteria lose their well-defined cell shapes (spheroplasts or L-forms ^10– 13^). At the same time, carefully isolated cell-wall sacculi retain the shape of the cell ^5, 14, 15^. While the cell wall is an elastic material that can undergo small changes of its surface area upon changes of turgor pressure (surface area can be stretched or compressed by about 5–20% in *Escherichia coli* ^16^), larger changes of morphology during growth require enzymatic remodeling of the cell wall. Specifically, existing bonds in the cell wall must be cleaved and new PG material must be inserted to avoid the formation of large holes that otherwise would lead to lysis ^17^. Regulation of cell-shape and cell-envelope integrity therefore requires the tight organization of cell-wall remodeling in space and time.

Bacteria organize the expansion of their cell walls in different ways. Some species add new cell wall exclusively at their poles, whereas others insert material in particular zones and yet others insert material at multiple (dispersed) locations along the long axis of the cell ^18, 19^. In this review, we will focus on the two best-studied model rods— *E. coli* and *Bacillus subtilis*—that display dispersed lateral cell-wall synthesis.

Although many of the molecular players involved in cell-shape regulation are known today ^20^, neither the physical mechanisms nor the regulatory cascades underlying different variables of shape (for example, cell diameter, cell length, and cell volume) are fully understood and they will be the subject of this review with a specific focus on rod shape.

Technological advances have made it possible to observe cell-wall synthesis in real time, either indirectly by following the dynamics of individual proteins involved in cell-wall expansion (for example, 7– 9, 21, 22) or directly by imaging the incorporation of fluorescently labeled D-amino acids into the PG wall ^23, 24^. These tools paired with novel techniques to perturb cell shape ^25, 26^ and the cell envelope-synthesizing machinery have revealed potential physical determinants of when and where cell-wall expansion happens. For example, experiments of physically bending cells have led to insights into the role of physical forces in shape regulation ^27– 29^. At the same time, advances have been made to connect cell shape with cell physiology at the level of both side-wall elongation ^30, 31^ and cell-division timing ^32– 37^. Here, we will highlight recent advances and open questions. We will first focus on the molecular and physical determinants of cell shape and then summarize newly identified and hypothesized connections between cell shape and cell physiology (in particular, metabolism and the chromosome replication cycle).

## The molecular and physical determinants of cell-wall remodeling

Changes of cell shape during growth require enzymatic remodeling of the cell wall ^3, 4, 20^. Proper cell-wall expansion, in turn, requires the coordination of multiple cytoplasmic and extra-cellular or periplasmic steps: (i) precursor synthesis and flipping from the cytoplasm to the extra-cellular/periplasmic side, (ii) cell-wall cleavage by DD-endopeptidases and possibly other hydrolases for cell-wall expansion along the long axis of the cell, (iii) glycan-strand polymerization (transglycosylation), and (iv) cross-linking to neighboring strands (transpeptidation). The PG cell wall is modified through other processes. For example, lytic transglycosylases cleave glycan strands, and PG material is taken out of the cell wall and recycled. The role of these processes might be to aid in the relaxation of mechanical stresses, but their mechanistic roles are still not understood. Most of the enzymes responsible for the different steps are known and described in great genetic and biochemical detail in previous reviews ^4, 20^. Here, we will discuss recent developments toward a better understanding of how the different processes of remodeling the cell wall are implicated in cell-shape regulation in rod-like bacteria, and we will focus on the model systems *E. coli* and *B. subtilis*.

### The role of synthesis and hydrolysis for cell-wall expansion

Physically, cleavage of the cell wall is strictly required for cell-wall expansion during growth. In agreement with this idea, endopeptidases have been found to be essential for Gram-negative *E. coli* ^38^ and *Vibrio cholerae* ^39^ and for Gram-positive *B. subtilis* ^40, 41^. Endopeptidases are thought to be particularly important, as they cut the bonds between neighboring glycan strands to allow the expansion of the cell wall along the long axis of the cell. Nevertheless, cell-wall cleavage has long been thought to be enslaved to cell-wall insertion in Gram-negative bacteria, where the cell wall is thought to be a thin layer ^5, 42, 43^. According to the “make-before-break” hypothesis, peptide bonds are cleaved only once a nascent glycan strand is ready for insertion. Höltje suggested that one glycan strand is cleaved out of the wall only after three new glycan strands have been connected to the neighboring strands—all performed by the same multi-enzyme rod complex (the 3-for-1 model) ^4^. Similarly, a recent study by Billaudeau *et al*. ^44^ suggests that three new glycan strands are added to two existing strands at the closest PG layer in *B. subtilis* ^8^. So far, co-localization of cell-wall synthases and endopeptidases has been observed only in *B. subtilis* ^45^ but not in Gram-negative bacteria. It is therefore also conceivable that cell-wall hydrolysis proceeds somewhat independently and possibly even upstream of cell-wall insertion ^38^. In Gram-positive *B. subtilis*, the cell wall is about 30 nm thick ^46, 47^. As new cell wall is added underneath the existing cell wall, old material must be cleaved at least partially at locations that are spatially disconnected from regions of synthesis. According to the “inside-out” model proposed by Koch and Doyle, new layers of PG are added underneath the cell wall while old outer layers are taken away by hydrolases ^48^. Thus, the coupling between hydrolysis and synthesis might be largely indirect ^46^, such that the turgor pressure drives PG cleavage, and PG synthesis is required merely to prevent the cell wall from thinning over time.

**Figure 1.**
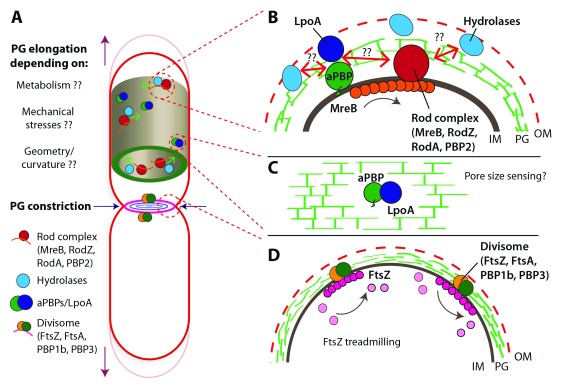
Schematic representation of the prevailing mechanisms of cell-wall synthesis in rod-shaped bacteria. ( **A**) Illustration of a rod-shaped cell indicating the rod complex, hydrolases, and class A penicillin-binding proteins (aPBPs) during cell growth (peptidoglycan [PG] elongation) and of the divisome during cell division (PG constriction). Despite decades of research on bacterial cell-wall growth, there are still many open questions about the factors that can actively influence PG assembly and cell growth, such as metabolism, mechanical stresses, and cell shape. ( **B**) Inset showing the major cell-wall synthesis machineries recently suggested to work partially independently ^21^: (i) the processively moving rod complex containing the transglycosylase RodA, the transpeptidase PBP2, and circumferentially oriented MreB filaments and (ii) the bi-functional aPBPs. Hydrolases may be actively engaged in cell-wall cleavage during cell-wall synthesis. Questions remain of how hydrolases interact with the rod complex or aPBPs or both. ( **C**) aPBPs are activated through outer membrane (OM) lipoproteins LpoA and LpoB. This interaction could provide a mechanism to sense pore sizes in the cell wall to direct PG synthesis. ( **D**) Division in rod-shaped bacteria is guided by treadmilling FtsZ filaments that are oriented along the circumference of the constricting cell. IM, inner membrane.

Recently, a long-standing paradigm for a single multi-enzyme machinery that inserts PG into the cylindrical part of rod-shaped *E. coli* and *B. subtilis* has been challenged. Instead, evidence suggests that two machineries contribute to side-wall synthesis somewhat independently ^21^ (see Figure 1B, C): on the one hand, the rod complex consisting of the mono-functional transpeptidase PBP2 (PBP2A and PBPH in *B. subtilis*) and the transglycosylase RodA in concert with the cytoskeletal MreB (the rod complex); on the other hand, the bi-functional class A penicillin-binding proteins (aPBPs) (importantly, PBP1a and PBP1b in *E. coli* ^21^ and PBP1, PBP2c, and PBP4 in *B. subtilis* ^49, 50^). The two types of machineries behave qualitatively differently and might have distinct functions. The rod complex moves around the cell in a persistent manner corresponding to processive insertion of long glycan strands ^7– 9, 21^, while the aPBPs move seemingly diffusively with intermittent pauses potentially corresponding to localized cell-wall insertion events ^21, 22^. Yet the machineries seem to largely depend on each other’s functionalities, as cell-wall synthesis is reduced by about 80% upon inhibition of either complex ^21^. It is thus possible that they work together and even form transient joint complexes. Elucidating the strengths and durations of these interactions will be important to fully understand the importance of the different components for cell-wall integrity and rod shape.

The Bernhardt lab recently found evidence for cell-wall synthesis through aPBPs partially being activated downstream of cell-wall cleavage in *E. coli* ^51^. Upon overexpression of the DD-endopeptidase MepS, cell-wall synthesis was found to increase and synthesis was ascribed largely to aPBP activity. Thus, aPBPs might sense the holes generated by DD-endopeptidases (see Figure 1B, C). We expand on the possibility of sensing pore size in the next section. Whether DD-endopeptidases can act fully independently of cell-wall synthesis will have to be seen in future experiments (for example, by reducing or inhibiting cell-wall synthesis and monitoring cell-wall hydrolysis).

### The role of mechanical forces for cell-envelope expansion

Prior to many molecular observations of cell-wall remodeling, Koch had suggested, in a pioneering discussion paper, that the rate of cell elongation is controlled by “smart autolysins” that cleave the PG cell wall in response to the buildup of mechanical stress in the cell wall in Gram-negative and Gram-positive bacteria ^42^. Stress in the cell wall, in turn, is due to the high intracellular turgor pressure. The pressures of about 0.3–3 atm in *E. coli* ^52, 53^ and 10 atm in *B. subtilis* ^54^ are caused by high concentrations of osmolites in the cytoplasm ^55^. Pressure-dependent cell-wall expansion has since been confirmed for different walled organisms, in particular for fission yeast ^56^ and plant cells ^57^ (see a recent review by Rojas and Huang ^58^). Very recent experiments by Rojas *et al*. in *B. subtilis* suggest that pressure also has an important role in regulating the elongation rate of Gram-positive bacteria ^59^. The authors found that a rapid reduction or a rapid increase of pressure through osmotic shocks reduces the rate of cell elongation and also the rate of cell-wall synthesis. A decrease of cell-wall synthesis upon hypoosmotic shock is likely caused by an increase in membrane tension and possibly by a tension-induced depolarization of the membrane. Rojas *et al*. suggest that the interplay among pressure, cell-wall stress, and membrane polarization guarantees that membrane surface area and cell-wall area remain coupled at all times. However, whether pressure is also responsible for coupling overall cellular biomass growth to the rate of cell-envelope expansion remains to be studied by assessing biomass growth and cell elongation independently. In the same realm, it will be interesting to elucidate how the reduction of pressure leads to a reduction of cell elongation mechanistically—through smart autolysins, through tension-dependent synthases as proposed by Rojas *et al*., or completely indirectly through changes of the overall growth rate of the bacteria. For the Gram-negative *E. coli*, the behavior is very different: the same authors showed, in a previous paper, that osmotic pressure was neither required nor responsible for cell-wall expansion on short time scales ^60^.

Despite the robustness of PG expansion in *E. coli* with respect to pressure, differential mechanical stresses in the cell wall have an impact on cell-wall expansion also in *E. coli*. Previous work has demonstrated that cells grow into curved or pancake-like shapes if physically bent or squeezed ^28, 61– 65^. In one of the first studies, Takeuchi *et al*. showed that rod-shaped *E. coli* can be subjected, confined, and adapted to various shapes upon antibiotic-induced filamentous growth ^28^. Recent studies by Amir *et al*. have demonstrated that cells fully recover from bent shapes: they straighten out at a rate almost twice as fast as their rate of elongation ^27, 65^. In quantitative agreement with a theoretical study of the mechanical stresses in the cell envelope during bending and straightening, the study suggests that both bending and straightening are due to asymmetric mechanical stress distributions in the cell wall ^27^. As a subtlety, Wong *et al*. ^27^ found that differential stresses along the long axis of the cell were too small to explain straightening. This is in agreement with previous work from Emonet *et al*. ^66^. However, they found relevant differences in stresses along the circumference of the cell, which could be responsible for differences in the pore size of the PG meshwork. An attractive hypothesis for the sensing of pore sizes in Gram-negative bacteria is that the aPBPs PBP1a and PBP1b together with their cognate outer-membrane lipoprotein activators LpoA and LpoB ^67, 68^ ( Figure 1C), respectively, sense the pore size in the bacterial cell wall ^20^ and thus insert more material at sites where pore sizes are increased because of mechanical stress. However, whether cell-wall synthases or hydrolases are responsible for shape changes upon mechanical forces and for the recovery to normal rod-like shape remains to be studied. **

### The role of the MreB cytoskeleton for rod-like cell shape

The model of stress-induced bending and straightening is in contrast to a previous geometry-based model by Ursell *et al*. ^69^. The bacterial cytoskeleton MreB localizes to the inner regions of bent cells ^64, 69^. Huang and colleagues ^69^ argued that the intrinsic curvature and twist of MreB filaments might be responsible for localizing MreB and thus the cell-wall synthesis machinery differentially to these regions. However, according to the more recent study by Wong *et al*. ^27^, differences in MreB localization were found to be too weak to account for the observed straightening dynamics. Instead, the MreB asymmetric localization observed could also be due to a combination of curved cell geometry and persistent circumferential motion ^27^, as previously discussed ^66^.

In the same study by Ursell *et al*., the authors found that MreB filaments are attracted to regions of negative Gaussian curvature and excluded from regions of positive Gaussian curvature, such as the cell poles ^69^, even in cells that are not bent. However, it remains to be studied how strong and important this effect is for the cylindrical part of the cell. In *E. coli*, the exclusion of MreB from the cell poles might be due to a curvature-independent, pole-specific effect: Kawazura *et al*. recently showed that the pole-specific enrichment for anionic phospholipids is required to exclude MreB filaments from cell poles ^70^.

The filamentous nature of the MreB cytoskeleton was long under debate ^71, 72^. Most recent measurements of fluorescent-protein fusions to MreB indicate that MreB forms independent filaments of a few hundred nanometers to a few micrometers in length in *E. coli* ^73, 74^ and *B. subtilis* ^75^ that are oriented circumferentially in both *E. coli* and *B. subtilis* ^73, 76^, with a slight chiral twist in *E. coli* ^73^. Interestingly, the orientation of MreB filaments seems to be directly coupled to the orientation of MreB tracks around the cell circumference and thus to the orientation of newly inserted PG material ^7, 73, 76^, in agreement with the macroscopic chiral organization of the cell wall ^29^. As MreB forms intrinsically curved filaments *in vitro* ^77^, curvature and potentially twist thus make MreB a strong candidate for independently mediating its own orientation inside the cell ^73, 78^. This picture is supported by a recent publication from the Garner and Löwe labs, which suggests that the intrinsic orientation of MreB is responsible for the circumferential orientation of MreB filaments and tracks in *B. subtilis* ^79^. They demonstrate that MreB filaments assume the direction of largest principal curvature if cell shape is imposed in micro-chambers, and they conclude that MreB’s track orientation is independent of any major local cell-wall structure. They therefore conclude that MreB’s intrinsic orientation solely determines the orientation of newly inserted glycan strands ^79^. The orientation preference of MreB might also be responsible for the exclusion of MreB from the poles: MreB filaments that move into the poles rapidly leave the poles again, while they are stabilized in the cylindrical part of the cell.

In *E. coli*, the picture could be more complicated. The correlations between MreB and cell-wall orientations could also result from a more subtle feedback loop between MreB’s filament properties and the properties of the cell-wall synthesis process. While MreB likely biases cell-wall insertion toward a near-circumferential orientation, the rod complex might also follow the existing glycan strands as a template for the insertion of new strands. The glycan-strand orientation, in turn, could come about due to a combination of MreB’s intrinsic orientation and chiral biases during cell-wall insertion. This hypothesis is in agreement with Huang *et al*., who showed that the chirality of MreB tracks can be affected by perturbations different from direct perturbations of MreB itself ^80^.

Ultimately, it seems reasonable to assume that the local properties of the cell wall and cell shape jointly determine where new cell-wall remodeling takes place. Jensen *et al*. recently suggested a numerical simulation of the cell wall on the basis of this principle that maintains cell shape over many generations of growth ^81^. The model showed that careful local organization of cell-wall synthesis based on the microscopic cell-wall geometry together with a bias of cell-wall insertion toward a circumferential direction is sufficient to maintain rod-like cell shape for many generations ^81^. Therefore, local interactions of enzymes with the cell wall together with the circumferential orientation sensed by MreB could be responsible for cell-wall integrity and cell-wall orientation. However, neither this nor previous models have established a physical determinant of cell diameter, and we simply do not yet understand how cell diameter can be highly adaptive to different growth environments but precisely maintained in cells growing in identical environmental conditions. We will come back to the determinants of cell diameter in one of the following sections.

### Cytokinesis and the septal ring

During cell division, both Gram-positive and Gram-negative rods ultimately form two hemispherical poles at midcell ^82^. The coordination of cell constriction and cell-wall synthesis is complex and requires the interplay of dozens of proteins ^82, 83^. A major determinant of the localization and orientation of the cell-division machinery is the tubulin homolog, FtsZ, together with accessory Zap proteins ^84^. FtsZ filaments are oriented circumferentially at midcell and thus are responsible for the circumferential orientation of glycan-strand insertion ^85^. FtsZ filaments were recently found to effectively move by treadmilling in *B. subtilis* and *E. coli* ^86, 87^ ( Figure 1D). Unlike the way in which MreB is driven around the cell by the rod complex, FtsZ treadmilling is responsible and rate-limiting for cell-wall insertion and septation in *B. subtilis* ^87^. In *E. coli*, treadmilling is required for proper cell septation, but the rate of septation is surprisingly reported not to be limited by FtsZ treadmilling ^88^.

FtsZ was previously argued to have a role in exerting a rate-limiting constrictive force during septation, based on structural data ^89^ and *in vitro* work ^90^. However, more recent work has questioned this function ^88^, as changes in the GTPase activity of the FtsZ ring did not significantly change the rate of constriction. On the contrary, perturbing the activity of major cell wall-modifying enzymes (notably, FtsI) led to changes of the rate of constriction. Therefore, it remains to be discovered how the dynamics of FtsZ and the cell-wall synthesis machinery jointly lead to the formation of two hemispherical poles at midcell.

## Cell shape, metabolism, and the cell cycle

One, if not the foremost, function of cell shape is to provide a well-defined volume for all intracellular processes required for the completion of every cell cycle. Empirically, average cell size is an exponentially increasing function of growth rate if growth rate changes because of nutrient limitation ^91– 93^. This fundamental “growth law” was first identified by Schaechter *et al*. ^91^. In rod-like bacteria, both cell diameter and average cell length contribute to the change in average cell volume. Yet the physical processes responsible for cell diameter and average cell volume are quite different: cell diameter is physically constrained by the cell wall. Accordingly, perturbations of cell-wall synthesis at the level of precursor synthesis, the rod complex, or the MreB cytoskeleton increase the cell diameter ^73, 80, 94^. During growth, the cell wall constantly expands to create space for the accumulation of intracellular biomass; hence, the rate of expansion must be tied to the rate of biomass growth. Average cell volume, on the contrary, is determined largely by the timing of cell division. Timing of cell division has often been ascribed to DNA replication ^33, 95, 96^, but recent studies suggest that the cell envelope could also play an important role in cell-size regulation ^30, 31^. Here, we discuss recent findings and remaining questions regarding both aspects of rod elongation and division and put particular emphasis on the potential positive regulatory roles of PG precursor production, PG hydrolysis, and membrane synthesis. For a more detailed account of upstream components that might be responsible for changes in precursor levels, see an excellent review by Sperber and Herman ^97^.

### The plasticity of cell diameter upon metabolic limitations of cell-envelope expansion

It was recently demonstrated by different labs that reducing cell-envelope capacity affects cell diameter in different ways ^30, 31, 98^. Most recently, Levin *et al*. showed that limitations of phospholipid synthesis jointly reduce cell diameter and cell length according to Schaechter’s growth law ^30^. Upon chemical perturbations of fatty-acid metabolism, cells produced fewer phospholipids, which led to reductions of both growth rate and cell size. Importantly, they also found that synthetic upregulation of lipid synthesis increased cell diameter, even if the growth rate was reduced. They thus proposed that membrane capacity was responsible for cell dimensions also under normal growth conditions. However, the mechanism responsible for the proposed coupling between lipid metabolism and the cell wall, which is ultimately responsible for cell diameter, remains to be identified. Levin *et al*. suggested that such coupling could happen indirectly through PG metabolism or through a common pathway component, undecaprenyl pyrophosphate. Following a similar logic, Harris and Theriot had previously hypothesized that both cell diameter and the timing of cell division could be governed largely by cell-wall metabolism ^31^.

Similarly to Levin *et al*., Harris and Theriot titrated the capacity of bacteria to build their cell envelope, here by using a chemical inhibitor of PG-precursor synthesis (fosfomycin) ^31^. Unlike in the perturbations of lipid metabolism, cells increased rather than decreased their cell diameter upon mild perturbations of PG availability while maintaining a constant growth rate. A similar observation was made by Dörr *et al*. ^99^ in *V. cholerae*: cells became wider or thinner upon a decrease or increase of cell-wall synthesis, respectively ^99^. Harris and Theriot argued that cells adjust their cell diameter to attain a desired ratio of surface area to volume (SA/V ratio), which is compatible with the limited resources of PG production. The SA/V ratio is governed predominantly by cell diameter and the natural quantity of interest under two interesting assumptions we will discuss further: first, the rate of PG synthesis per total cytoplasmic proteome is given by the physiological state of the cell and is limiting for the rate of cell-wall expansion. Second, the density of PG per cell-wall area is constant and is based on the conservation of this quantity both under normal growth conditions and upon perturbations of the MreB cytoskeleton ^100^. Under these two assumptions, the SA/V ratio is fully determined by the rate of PG synthesis per cytoplasmic volume and the density of PG per cell-wall area. But as a complication and challenge to the simple model, the SA/V ratio changes during the cell cycle. The ratio decreases after birth and then increases toward division because of the formation of a septum. To rescue the model, Harris and Theriot hypothesized a pool of PG precursor material that buffers changes of PG synthesis during the cell cycle. Although the model is generally attractive in that it postulates diameter control on the basis of precursor metabolism rather than mechanical details of cell-wall insertion and the cytoskeleton ^101^, the mechanistic implementation of the different proposed roles of PG for the regulation of cell diameter and cell division remains to be discovered.

As an alternative to the hypothesis for cell-shape regulation through precursor production, cell-wall expansion could be controlled at the level of cell-wall cleavage, specifically through the hydrolysis of PG bonds, as already indicated above. Redundantly essential sets of DD-endopeptidases have been identified in different model bacteria ^38, 41^. Thus, the regulation of cell-wall hydrolases is highly important for the maintenance of rod-like shape. Accordingly, variations in hydrolase activity through substrate modification ^102^ or through changes of hydrolase abundance lead to changes in cell shape ^103^. Thus, it is conceivable that the rate of cell-surface expansion is regulated through hydrolase activity while cell-wall synthesis would be responsible merely for maintaining cell-wall integrity.

The studies by Levin *et al*. and Theriot *et al*. provide valuable insights into the plasticity of cell diameter upon perturbations of different aspects of cell-envelope expansion. While each of the studies proposes a single pathway of cell-envelope biogenesis to be responsible for cell diameter under normal growth conditions, it is also conceivable that fluxes or pool sizes of PG precursors and phospholipids, together with other regulatory components, jointly determine cell diameter during regular growth. Indeed, the surface areas of cell wall and cell membranes are almost equal during normal growth ^43^. Their synthesis is therefore likely coordinated. Coordination could happen both on a metabolic and on a physical level. As already indicated above, changes of membrane metabolism could feed back on PG metabolism (for example, through the regulatory molecule ppGpp). Physically, cell wall and membranes are coupled in multiple ways: as already discussed above, mechanical stress in the cell membrane could serve to couple cell-wall expansion to membrane synthesis ^59^. Previous work by Ehlert ** and Höltje ^104^ demonstrated that membrane synthesis is required for the flipping of PG precursor material, providing yet another way of coupling the two envelope components. Furthermore, many cell-wall remodeling enzymes are embedded in the cytoplasmic membrane and thus could serve as sensors of the local distance between lipid membrane and cell wall.

Together, cell diameter and the rates of membrane and PG synthesis could be limited by multiple pathways and could feed back on each other. This cycle would be controlled analogously to other processes such as overall growth rate, which is determined and limited simultaneously by different processes, in particular through ribosome levels, amino acid concentration, and nutrient availability ^105, 106^. Determining causality and identifying the role of each component for diameter control under normal growth conditions will likely require a more mechanistic understanding of their respective contributions.

### Average cell size is governed by the timing of cell division

Contrary to Schaechter’s growth law, if the growth rate is reduced because of the excess production of unnecessary proteins, cell size increases with decreasing growth rate ^93^. Both Schaechter’s growth law and the inverse behavior are intuitive. A cell requires a larger number of ribosomal and metabolic proteins at fast growth compared with slow growth to complete a larger set of tasks in a shorter time. The production of unnecessary proteins requires space for the protein itself and for additional proteins and ribosomes devoted to their production. Although these relationships are intuitive, we still have not understood the regulatory mechanisms underlying cell size control in different physiological conditions, and their discovery is a very active field of research ^37^. Following the establishment of Schaechter’s growth law ^91^, Donachie ^107^ and Helmstetter *et al*. ^108^ found a striking relationship between chromosome replication and cell size that since has served as a major motivation to study DNA replication as a candidate for the regulatory process upstream of size control: Donachie found that cells initiate new rounds of DNA replication on average at a well-defined cell size that is independent of growth rate ^107^. This study was recently extended and confirmed for many alternative ways of changing growth rate, cell size, and the replication cycle ^109^.

From a physiological point of view, it makes sense that DNA replication serves as a checkpoint for cell division (that is, that cells divide only once every daughter cell has inherited one complete chromosome). Accordingly, cells with a reduced rate of DNA replication show a larger average cell size. However, alternatively, it is also possible that there are other checkpoints for cell division that might be limiting depending on physiological conditions. Harris and Theriot suggested that cells accumulate the PG material required for cell septation during the course of a cell cycle and that this “excess material” is responsible for the timing of cell division ^31^. Levin *et al*. suggested that membrane capacity and carbon catabolism limit the time of cell septation ^30, 110, 111^. Similarly to the question of what determines cell diameter, it remains to be discovered which of the processes is limiting under which conditions.

A powerful method to identify causal relationships between different cell cycle-related quantities is to study correlations at the single-cell level. With this approach, bacteria and eukaryotes were recently found to divide after adding a well-defined volume to their cell size at birth, as opposed to dividing at a predetermined cell size (see, e.g., 33, 93). Whether the adder principle is a result of coupling cell division to replication or whether there is an independent mechanism coupling cell division to cell size or added cell size will have to be studied in the future.

## Conclusions

Recent experiments and theoretical models have led to interesting new findings and hypotheses about the determinants of cell shape in rod-like bacteria, of which we could present only a subset in this review because of space constraints. We can now observe the behavior of the microscopic machinery responsible for cell-wall remodeling at an unprecedented resolution at the single-cell level. At the same time, genetics, biochemistry, and structural biology provide insights into candidate regulators and modulators that can be tested for their physical role in cell-shape regulation. Thus, coarse-grained physical models of cell shape can now be related to the action of specific proteins. At the same time, we can observe aspects of cellular physiology, such as growth rate, metabolism, and energetic state, with increasing resolution and accuracy. We can thus relate the physics of cell shape to other physiological observables by using coarse-grained descriptions of regulatory interactions and identifying specific molecular pathways responsible for the interactions.

Whereas the last 30 years have focused largely on the role of individual genes and proteins for cellular physiology in isolation, the coming years will likely provide a plethora of insights into their actual physical role in the cell by taking the response of the cell into account. We will thus likely obtain answers to questions that were often raised decades ago on the basis of empirical relationships such as Schaechter’s growth law of cell size or the Donachie relationship between DNA replication and division. Yet many challenges remain, as causative relationships have to be deduced from correlations and as the potential interactions between different modules of the cell are almost countless. An important role will thus be played by mathematically and physically modeling the cell at different levels of resolution. Very successful examples of such models can be found in the study of cellular growth ^112– 114^ and the cell cycle ^115^.
